# Social success in a noisy world: exploring the relationship between decreased sound tolerance and social profiles

**DOI:** 10.3389/fpsyg.2025.1560100

**Published:** 2025-05-22

**Authors:** Ashleigh Wickie, Natalia Van Esch, Nichole E. Scheerer

**Affiliations:** Deparment of Psychology, Wilfrid Laurier University, Waterloo, ON, Canada

**Keywords:** decreased sound tolerance (DST), social competence, sensory processing, misophonia, hyperacusis

## Abstract

Humans are inherently social creatures, yet considerable variability exists in our social behaviours. It is unclear what factors contribute to this variability. Given the complex and abundant sensory stimuli present in our daily environments, differences in sensory processing abilities may contribute to the variation observed in social behaviours. Individual differences in sensory processing may have significant effects on an individual’s capacity to navigate social settings and may influence the development and expression of social competence. Existing literature also suggests that it is common for individuals with one form of sensory processing difference, Decreased Sound Tolerance (DST), to engage in social avoidance behaviours to mitigate exposure to distressing sounds. However, limited research explores the potential relationship between DST severity and social competence. Therefore, this study investigated the relationship between DST and social competence. As such, a sample of 2095 undergraduate students completed an online survey designed to assess their DST severity and social competence. Initially, to parse the variability in social competence, scores on the multidimensional social competence scale (MSCS), underwent a k-means cluster analysis. This analysis yielded four unique social profiles based on seven social competence domains (e.g., social motivation, emotion regulation etc.). Misophonia and hyperacusis questionnaires were then used to evaluate differences in DST across the social profiles. The results indicated varying severity levels of both misophonia and hyperacusis across the four social profiles, with the individuals who reported the highest social competence exhibiting the lowest levels of DST. These findings highlight the potential relationship between sensory processing differences, such as DST, and social functioning.

## Introduction

Social interactions play a pivotal role in our everyday experiences as humans. Social characteristics and behaviors like empathy, assertiveness, self-monitoring, and sociability can influence the quality of social interactions (Riggio, 1986). However, social abilities are extremely variable across individuals, and limited information is known about the factors that contribute to the heterogeneity in the development and expression of social competence. Sensory processing differences have been demonstrated to be predictive of social abilities in both autistic (Hilton et al., 2007; Kose et al., 2023; Scheerer et al., 2021) and non-autistic (Kose et al., 2023) individuals. As such, these predictive relationships suggest that sensory processing differences may be a potential factor contributing to individual differences in social abilities. The aim of this current research is to parse the variability in social abilities by modeling social profiles. By comparing decreased sound tolerance (DST), a common sensory processing challenge, across these social profiles, we may gain further insight into the relations between sensory and social processing.

DST is broadly defined as a reduced tolerance of everyday sounds (Williams et al., 2021; Cash, 2015). Two common subtypes of DST are misophonia and hyperacusis. Misophonia is characterized by strong negative emotional, physiological, and behavioral responses to “trigger” sounds that are not observed in most people (Swedo et al., 2022). Hyperacusis, on the other hand, is a reduced tolerance to sound(s) at levels that are not bothersome to the majority of other people (Adams et al., 2020). DST has been linked to functional impairments. Specifically, across both child (Scheerer et al., 2021) and adult (Dixon et al., 2024; Scheerer et al., 2024a,b,c; Williams et al., 2021) samples, DST has been shown to negatively influence one’s ability to participate in activities at home, at school, and in the community. Further, DST has been associated with increased anxiety and depression and a reduced quality of life (Cash, 2015; Yilmaz et al., 2017; Scheerer et al., 2024a,b,c). Information regarding the prevalence and individual differences that are predictive of DST are only beginning to emerge. DST has been demonstrated to be more likely to occur in individuals affected by tinnitus (Cash, 2015). DST is also commonly present in autistic individuals and is often the most prevalent and disabling sensory characteristic of autism (Williams et al., 2021). Additionally, a recent misophonia prevalence study has suggested that females, along with younger individuals (under 55 years of age), those with less than a high school education, those who never married, and those with lower income experience more severe misophonia symptoms (Dixon et al., 2024). Evidence has also suggested that chronic misophonia symptoms surfacing in childhood and adolescence continue into adulthood (Dixon et al., 2024). Given that DST has been linked to the avoidance of social environments, it is possible that the social avoidance caused by DST may influence the development and expression of social competence.

The heterogeneity in the development and expression of social skills across individuals can complicate the investigation of relations between social processing differences and other potentially related factors, such as sensory processing abilities. One way to circumvent this, is to parse the variability in social skills by creating social profiles. The identification of social profiles has functional and clinical applications. If patterns of social abilities reliably co-occur across individuals, these profiles may facilitate the identification of other characteristics that tend to co-occur with these different social profiles. For example, individuals with a particular social profile may tend to have more extreme sensory processing differences or a greater likelihood of having or developing DST. Therefore, the first aim of the current study is to parse the social competence heterogeneity to investigate whether patterns of social abilities exist within a large sample of adults. The Multidimensional Social Competence Scale (MSCS) was developed to be used as a standardized measure of social abilities (Yager and Iarocci, 2013). The MSCS includes seven distinct social competence domains, including social motivation (SM), social inferencing (SI), demonstrating empathic concern (DEM), social knowledge (SK), verbal conversation skills (VCS), nonverbal sending skills (NSS), and emotion regulation (ER; Yager and Iarocci, 2013; Trevisan et al., 2018). The MSCS was developed initially to assess the variability in social expression and the severity of social challenges demonstrated by autistic individuals, as social difficulties were highlighted as the most distinctive and defining characteristic of autism. However, extreme variability in social abilities is witnessed across the autism spectrum (Yager and Iarocci, 2013). The 2013 study developed a parent-report version of the MSCS for assessing adolescents (Yager and Iarocci, 2013). However, more recently, a psychometric evaluation of the MSCS for young adults was conducted to validate the self-report version of the MSCS for both autistic and non-autistic adults (Trevisan et al., 2018). This validation provides significant support for the reliability of the self-report version of the MSCS for both autistic and non-autistic individuals.

As previous links have been established between social and sensory processing (Hilton et al., 2007; Kose et al., 2023; Scheerer et al., 2021) and DST has been shown to promote social avoidance, the second aim of the current study was to investigate a potential relationship between DST and social competence. To this end, the Duke Misophonia Questionnaire (DMQ; Rosenthal et al., 2021) and the Inventory of Hyperacusis (IHS; Greenberg and Carlos, 2018) were used to evaluate misophonia and hyperacusis, respectively. The DMQ was developed to thoroughly assess the severity, negative impact, and methods of coping with misophonia (Rosenthal et al., 2021), while the inventory of hyperacusis symptoms (IHS) scale was developed to evaluate the presence and severity of hyperacusis symptoms (Greenberg and Carlos, 2018). By comparing these measures across MSCS social profiles it was possible to explore relations between DST severity and social competence. It was expected that increased DST symptom severity would be associated with social profiles characterized by increased social difficulties. Given neither misophonia nor hyperacusis are currently recognized by the Diagnostic and Statistical Manual of Mental Disorders (DSM-5 TR; American Psychiatric Association, 2022), despite their significant levels of impairment, identifying relations between DST and social competence may help to better define the broader consequences of DST beyond intolerance to sound. As such, this research may help to emphasize the need to support individuals with DST in providing accommodations and mitigating the adverse effects of DST on social competence development and expression.

## Methods

### Participants

A sample of 2095 undergraduate students from Wilfrid Laurier University ranging in age from 18 to 60 years were recruited to participate in this study (see Table 1 for full demographic information). Participants were compensated with course credit. Study procedures were approved by the Research Ethics Board at Wilfrid Laurier University and are in accordance with the World Medical Association’s 2013 Declaration of Helsinki.

**Table 1:** Complete demographic information and questionnaire scores for the sample, including mean, standard deviation, range, and the sample size for age, gender, decreased sound tolerance (DST) scores and multidimensional social competence scale (MSCS) total and subscale scores.

Demographic factor	n	Mean	SD	Range	Reliability Cronbach’s ɑ
Gender					
Male	436				
Female	1,614				
Other (see below)	45				
Age	2059	20.34	2.76	18–60	
DMQ Severity	2095	22.16	19.67	0–92	0.98
DMQ Coping	2095	21.34	17.85	0–84	0.98
IHS Total Score	2095	39.94	15.40	0–100	0.96
MSCS Total Score	2095	286.56	31.29	130–381	0.94
Social Motivation	2095	37.54	7.47	13–55	
Social Inferencing	2095	41.04	6.24	15–55	
Displaying Empathic Concern	2095	43.72	6.38	18–55	
Social Knowledge	2095	46.59	5.77	11–55	
Verbal Conversation Skills	2095	38.40	6.77	12–55	
Nonverbal Sending Skills	2095	42.07	6.34	13–55	
Emotion Regulation	2095	37.19	6.06	14–55	

DMQ, Duke Misophonia Questionnaire; IHS, Inventory of Hyperacusis Symptoms; MSCS, Multidimensional Social Competence Scale. “Other” includes genders: other (*n* = 3), prefer not to say (*n* = 7), transgender (*n* = 11), nonbinary (*n* = 20), I do not know (*n* = 1), gender fluid (*n* = 2), and gender binary (*n* = 1).

### Materials

#### Duke misophonia questionnaire

The DMQ (Rosenthal et al., 2021) is an 86-item questionnaire used to measure misophonia severity across nine subscales. Responses are made using a Likert scale that ranges from 0 (“Never”) to 4 (“Always/Almost Always”). The DMQ yields a Symptom Severity Composite Score and a Coping Composite Score, comprised of the subscales: Trigger Frequency, Affective Responses, Physiological Responses, Cognitive Responses, Coping Before (triggered), Coping During (triggered), Coping After (triggered), Impairment, and Beliefs. The Symptom Severity Composite Score is a combination of subscales measuring cognitive, affective, and physiological responses across 23 items to measure overall symptom severity. This composite measures how often participants thought/felt/responded in a particular manner when exposed to trigger sounds (e.g., “I want to cry,” “I felt hostile,” “My heart pounded or raced,” respectively). Higher Symptom Severity Composite scores indicated more severe symptoms were experienced when exposed to trigger sounds. The Symptom Severity Composite Scale has a maximum score of 96. In this current study, to achieve the threshold for clinical misophonia, participants were required to have a mean item score of 1.8 or higher, or a total mean score of 41.4 or higher on the Symptom Severity Composite (Rosenthal et al., 2021). Additionally, the 21-item Coping Composite Score measures coping before, during, and after exposure to trigger sounds. Various items were used to measure coping prior (e.g., “I used a different sound to drown out bothersome sounds”), during (e.g., “I produced an alternate sound”), and after (e.g., “I listened to a comforting sound”) exposure to trigger sounds. Higher Coping Composite scores indicated a greater use of coping strategies and more effective coping skills overall. The DMQ has good marginal reliability for both coping composite score and symptom severity score (p_xx_ = 0.93; p_xx_ = 0.93; Rosenthal et al., 2021). Additionally, high correlations were found between the Misophonia Assessment Questionnaire (MAQ), Misophonia Emotional Response Scale (MER), Misophonia Coping Responses Survey (MCR), and the Amsterdam Misophonia Scale (A-MISO-S) and the DMQ Symptom composite score (correlation coefficients ranging from *r* = 0.72 to *r* = 0.81), and these correlations between the DMQ Symptoms scale and already existing measures were used to determine preliminary convergent validity for the DMQ Symptoms Severity Composite Score (Rosenthal et al., 2021). Existing literature (Rosenthal et al., 2021) has demonstrated that the DMQ Symptom Severity scale scores demonstrate a good ability to differentiate between clinical and non-clinical thresholds of misophonia, by comparing those who score higher than 7 (clinical) and lower than 7 (non-clinical) on the misophonia questionnaire (MQ; Wu et al., 2014; *AUC* = 0.82, 95% CI [0.77–0.87]). Cronbach’s alpha for the current study indicates reliability was excellent *α* = 0.98.

#### Inventory of hyperacusis symptoms scale

The IHS (Greenberg and Carlos, 2018) is a 25-item questionnaire used to measure hyperacusis severity, assessing loudness perception, negative emotions, fear, cognitive, social, occupational, and psychological functioning, as well as quality of life and mental health. The response options are on a 4-point Likert scale ranging from 1 (“Not at all”) to 4 (“Very much so”). The items measure the frequency/intensity of experiences (e.g., “I find the challenges of being exposed to loud sounds can make it difficult to do the things I used to enjoy”) when exposed to trigger sounds. The IHS is scored out of 100, where higher total scores indicate greater severity of hyperacusis symptoms. The IHS was developed and validated using a sample of 324 non-autistic adults with varying levels of auditory sensitivity. In the original introduction to the IHS, a clinical cutoff score of 75 was used (Greenberg and Carlos, 2018), however, a more recent study was conducted assessing the internal consistency and convergent validity of the IHS in a clinical population (Aazh et al., 2021). This recent study suggested that a cutoff score of 56 yielded more accurate results for classifying patients correctly. Therefore, for the current paper we used a cutoff score of 56 or greater to indicate clinical levels of hyperacusis severity. The IHS has high internal consistency and reliability (Cronbach’s *α* = 0.93; Greenberg and Carlos, 2018; Cronbach’s α = 0.96, current study) as well as significant convergence with measures of mental health and well-being, specifically the Patient Health Questionnaire-4 (PHQ-4) and the World Health Organization Brief Quality of Life Scale (WHOQOL-BRIEF; Scheerer et al., 2024a,b,c).

#### Multidimensional social competence scale

The MSCS (Trevisan et al., 2018) is a 77-item questionnaire used to measure social competence. The MSCS uses a 5-point Likert scale, and response options for each item range from 1 (“Not True/Almost Never True”) to 5 (“Very True/Almost Always True”). The items on the MSCS assess social competence across seven subscales, including social motivation (e.g., “I prefer to spend time alone”), social inferencing (e.g., “I recognize when people are trying to take advantage of me”), demonstrating empathic concern (e.g., “I am sensitive to the feelings and concerns of others”), social knowledge (e.g., “I know about the latest trends for my age”), verbal conversation skills (e.g., “I dominate conversations so that it can be hard for others to get a word in”), nonverbal sending skills (e.g., “My facial expressions seem ‘flat’”), and emotion regulation (e.g., “I have ‘meltdowns’”). As such, the MSCS allows individual differences in social competence, as well as more specific strengths and challenges related to one’s social abilities to be identified. Higher MSCS scores across the seven domains and a higher MSCS total score indicate greater social competence. Among a sample of adults, the MSCS was previously found to have good internal consistency and reliability (Cronbach’s *α* = 0.80; Trevisan et al., 2018). In the current study the MSCS was found to have excellent reliability α = 0.94.

### Procedure

Participants were recruited over an 8-month period from Wilfrid Laurier University’s research participation system and the survey was administered online via Qualtrics. Following informed consent, participants completed a survey that collected demographic information (e.g., birth date, gender, ethnicity, etc.), followed by a series of questionnaires, including the Beck Depression Inventory (BDI; Beck et al., 1961), Duke-Vanderbilt Misophonia Screening Questionnaire (DVMSQ; Williams et al., 2022), Beck Anxiety Inventory (BAI; Beck et al., 1988), Misophonia Questionnaire (MQ; Wu et al., 2014), Adult/Adolescent Sensory Profile (AASP; Brown and Dunn, 2002), Autism Spectrum Quotient (AQ; Baron-Cohen et al., 2001), Duke Misophonia Questionnaire (DMQ), Inventory of Hyperacusis Symptoms (IHS), and the Multidimensional Social Competence Scale (MSCS). However, for the purpose of this study, only the data from the MSCS, DMQ, and IHS will be reported as they align most closely with our research objectives. The remaining data, while informative, are outside the scope of the current analyses and will be addressed in future manuscripts. After completion of the survey, participants were then debriefed and compensated for their participation with course credit.

### Data analysis

The data were analyzed using Jamovi (The jamovi project, 2024), the Statistical Package for the Social Sciences (SPSS) software—version 28 (IBM Corp, 2023), and R Statistical Computing software (R Core Team, 2023). Given the large sample size (*n* = 2095) normality was assessed visually using q-q plots. All variables, except for age, appeared normal thus parametric tests were used. For age, non-parametric tests were used. Additionally, in instances where Levene’s test (Levene, 1960) indicated that the assumptions of homogeneity of variances were violated, Games-Howell post-hoc tests (Howell, 2010) were used.

First, we ran correlational analyses between all MSCS subscales and both DMQ and IHS scores to assess if a relationship existed between DST and social competence. As DMQ and IHS scores violated assumptions of normality, we ran Spearman’s rho. Given the significant correlations, we then moved forwards with running our cluster analysis.

To begin, participants’ MSCS subscale scores were converted to z-scores and subjected to a k-means cluster analyses using the k-means function in R (version 4.5.0) to identify patterns of social abilities. A cluster analysis is an exploratory data analysis technique that can be used to identify subgroups (or clusters) in a dataset that represent participants that are like one another, but distinct from other participants in other clusters. With a k-means cluster analysis, the algorithm groups the participants into a predefined number (k) of non-overlapping clusters. Here, participants were assigned to a particular cluster in such a way that the sum of the squared distance between all the participants MSCS scores, and the mean of all participants’ MSCS scores that belong to a particular cluster, was minimized (Hartigan and Wong, 1979). Using this approach with the MSCS data allowed us to examine how social abilities cluster together, with each of the resulting clusters representing a distinct social profile. For this analysis, subscales scores, rather than latent factor scores, were analyzed for ease of interpretability. To determine the model with the best fit, we used Bayesian Information Criteria (BIC; Zhong and Ghosh, 2003), within-cluster sum of squares (WCSS; Thinsungnoena et al., 2015), silhouette analysis (Thinsungnoena et al., 2015), and considered the unique explanatory power provided by each solution such that redundancies across clusters were minimized. Combining objective (e.g., BIC, WCSS) and subjective assessments balanced the statistical fit of the models with their potential to have practical, real-world significance.

Second, a comparison of the DMQ symptom severity composite scores, DMQ coping composite scores, and IHS total scores was conducted across the resultant social competence profiles via a one-way analysis of variance (ANOVA). The DMQ symptom severity composite scores, the DMQ coping composite scores, and the IHS total scores were treated as three distinct dependent variables, and the independent variable was the social profiles identified through the cluster analysis. The purpose of this ANOVA was to determine whether DMQ severity, DMQ coping, and IHS total scores were significantly different across the social competence profiles. Games-Howell post-hoc analyses were used to determine how DST severity varied across the social profiles. Since assumptions of homogeneity of variance, as assessed with Levene’s test, were violated for all dependents, we employed Games-Howell corrections when completing pairwise comparisons during post-hoc.

Given the large sample size used in this study, effect sizes of partial eta-square (*ηp*^2^ > 0.06), Cohen’s d ≥ 0.20, Cramer’s V > 0.20, and McFadden R^2^ > 0.20, were used as the threshold for all statistical analyses as these represent the cutoffs for moderate effects. As such, anything above this value should indicate a meaningful effect (Cohen, 1994; Lang et al., 1998; Sullivan and Feinn, 2012; Scheerer et al., 2024a; Scheerer et al., 2024b; Scheerer et al., 2024c; Tomczak and Tomczak, 2014).

## Results

Preliminary first-order correlations were run with each MSCS subscale, assessing how each subscale related to DMQ Symptom Severity, Coping, and IHS total score. As DMQ and IHS scores violated assumptions of normality, we ran Spearman’s rho correlations. All MSCS subscales other than DEM were significantly (*p* < 0.001) negatively related to all DMQ and IHS scores (see Supplementary Table S1 for exact rho values).

### Cluster analysis: patterns of social competence

**Figure 1:**
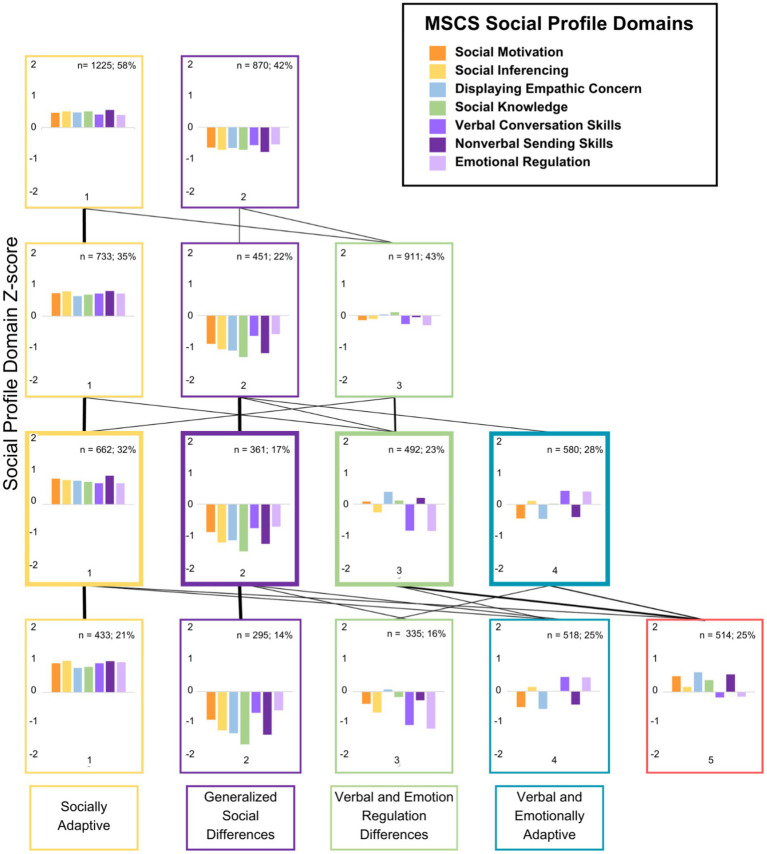
Social competence profiles based on z-scores across the k 2–5 cluster solutions. Negative z-scores indicate increased social difficulties, and the line weights between cluster solutions represent the number of participants changing clusters across the different solutions.

Results of the k-means cluster analyses indicated that a four-cluster solution produced the best fit after considering BIC values, within-cluster sum of squares (WCSS), and silhouette analyses (see Supplementary Figure S1) and the unique explanatory power of the social profiles. The first analysis used a *k* of 2. The two-cluster solution differentiated those with high social competence from those with low social competence, relative to the group mean (see Figure 1). With the addition of a third cluster, a group marked with difficulties with Verbal Conversation Skills and Emotional Regulation emerged. Upon the addition of a fourth cluster, we observed a group with highly adaptive Verbal Conversation Skills and Emotional Regulation, but relative difficulties with Social Motivation, Demonstrating Empathic Concern, and Non-Verbal Sending Skills. With the addition of each cluster, the model produced a new social profile that highlighted a distinct pattern of social competence. However, once the five-cluster solution emerged, the new cluster did not produce a highly differentiated pattern of social competence. As such, the *k* 4 cluster solution was selected (see Figure 1). Cluster one was classified as a socially adaptive (SA) profile, cluster two as a generalized social differences (GSD) profile, cluster three as a verbal and emotional regulation differences (VED) profile, and cluster four as a verbal and emotional regulation adaptive (VEA) profile.

**Figure 2:**
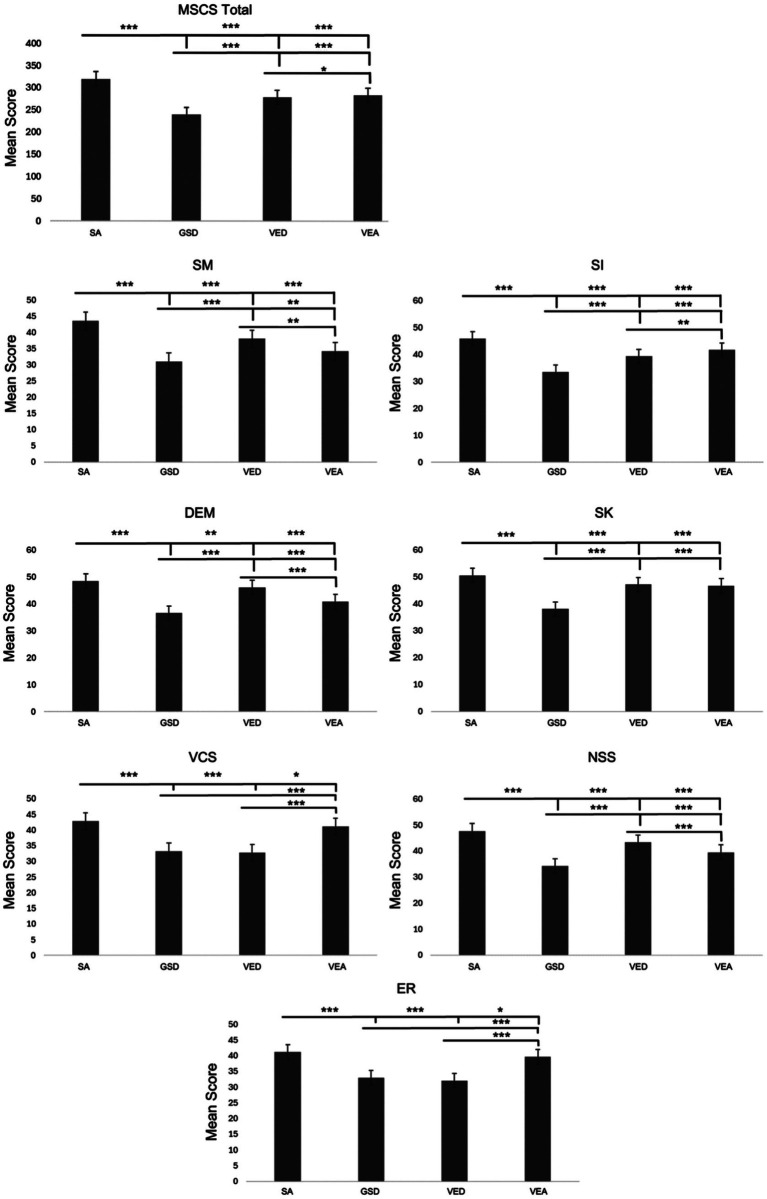
Social profile domain average scores across the four social profiles: social adaptive (SA), generalized social differences (GSD), verbal and emotion regulation differences (VED), and verbal and emotionally adaptive (VEA) for each of the seven subscales of the multidimensional social competence scale (MSCS): social motivation (SM), social inferencing (SI), demonstrating empathic concern (DEM), social knowledge (SK), verbal conversation skills (VCS), non-verbal sending skills (NSS), and emotional regulation (ER). Error bars indicate standard error of the mean. The bars above indicate differences between social profiles with each line using a different social profile as the reference group. The magnitude of the effect is denoted with asterisks identifying comparisons that are of a moderate effect size or larger, *d ≥ 0.2, **d ≥ 0.5, ***d ≥ 0.8.

One-way ANOVAs were conducted on the MSCS sub-scale scores (z-scores: see Figure 1, raw scores: see Figure 2) using JAMOVI to examine if the MSCS subscales varied across the four profiles. A main effect of social profile indicated that MSCS subscale(s) differed significantly across the social profiles (*ηp*^2^ > 0.06). *Post hoc* tests indicated that MSCS subscales varied across each social profile, except, social knowledge which was not significantly different between the VED and VEA social profiles (*d* < 0.2), and both verbal conversation skills and emotion regulation which were not significantly different between GSD and VED social profiles (*d* < 0.2; see Figure 2).

### Demographic factors

Table 2 reports the descriptive statistics and results regarding relevant demographic information, as well as the means and standard deviations of the exploratory measures for each social profile. Means of the DMQ symptom severity composite score, DMQ coping composite score, IHS total score, and MSCS total score are reported for each social profile, as well as age and gender.

**Table 2:** Demographic information, including means and standard deviations of the experimental measures for each social profile.

Factor	SA	GSD	VED	VEA
n	662	361	492	580
Age	21.1 (4.7)	20.6 (8.4)	19.1 (3.3)	19.6 (1.4)
Gender*	92 (M) 565 (F) 5 (O)	96(M) 245(F) 20(O)	43(M) 435(F) 14(O)	205(M) 369(F) 6(O)
DMQ Severity***	15.2 (16.0)	29.3 (22.0)	31.5 (20.5)	17.8 (16.5)
DMQ Coping**	16.4 (16.9)	26.4 (18.2)	27.8 (18.2)	18.3 (15.9)
IHS Total***	34.2 (10.7)	47.1 (18.3)	46.4 (16.6)	36.6 (13.0)
MSCS Total***	320 (14.8)	240 (18.4)	279 (14.9)	284 (13.4)
Social Motivation***	43.6 (5.5)	31.1 (5.9)	38.1 (6.0)	34.2 (6.0)
Social Inferencing***	45.8 (4.4)	33.5 (5.12)	39.4 (4.8)	41.7 (4.4)
Displaying Empathic Concern***	48.4 (3.9)	36.5 (5.3)	46.1 (4.5)	40.8 (5.0)
Social Knowledge***	50.6 (2.9)	38.1 (6.0)	47.2 (3.6)	46.7 (3.5)
Verbal Conversation Skills***	42.9 (4.9)	33.3 (5.8)	32.8 (5.4)	41.2 (4.5)
Nonverbal Sending Skills***	47.7 (3.8)	34.2 (4.7)	43.3 (4.2)	39.5 (4.2)
Emotion Regulation***	41.2 (4.4)	32.9 (4.9)	32.1 (4.7)	39.6 (4.3)

Standard deviations are indicated in brackets. The key demographic information and means and standard deviations of experimental measures were reported. *V > 0.20, **ηp^2^ ≥ 0.06, ***ηp^2^ ≥ 0.14 partial eta squared, indicating factors that varied by moderate effect sizes or larger across the four social profiles. DMQ, Duke Misophonia Questionnaire; IHS, Inventory of Hyperacusis Symptoms; MSCS, Multidimensional Social Competence Scale. Genders are noted as Male (M), Female (F) and Other (O), with “Other” including genders: other (*n* = 3), prefer not to say (*n* = 7), transgender (*n* = 11), nonbinary (*n* = 20), I do not know (*n* = 1), gender fluid (*n* = 2), and gender binary (*n* = 1).

#### Age

A Kruskal-Wallis test indicated that age was not significantly different across the four social profiles, χ^2^ (3) = 3.03, *p =* 0.39.

#### Gender

**Figure 3:**
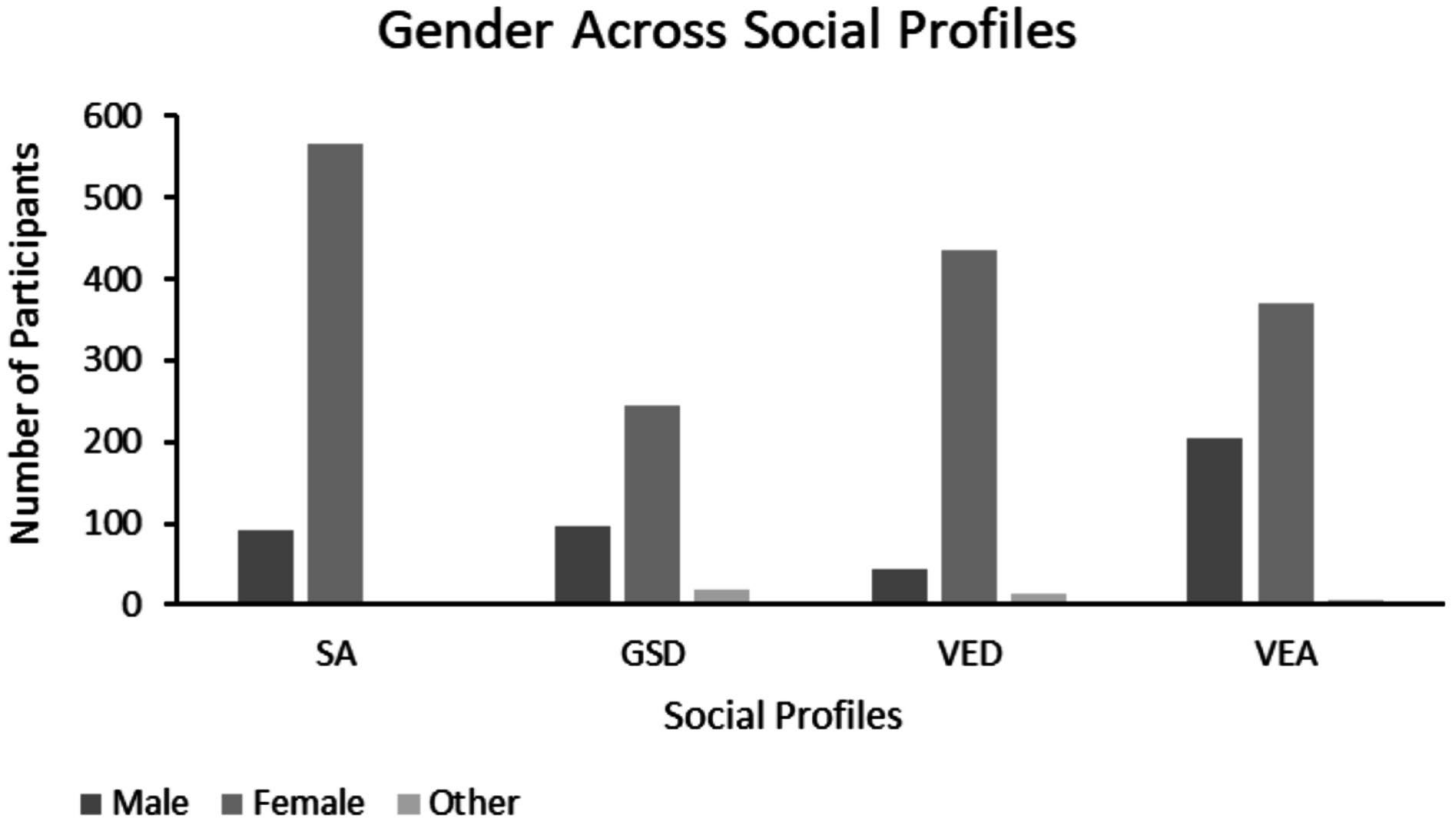
Gender across the social profiles. Left to right: male, female, and other. “Other” includes genders: other (*n* = 3), prefer not to say (*n* = 7), transgender (*n* = 11), nonbinary (*n* = 20), I do not know (*n* = 1), gender fluid (*n* = 2), and gender binary (*n* = 1).

A chi-square test of independence was conducted to investigate the relationship between gender and social competence profile. Gender significantly differed across our four social profiles (Figure 3), χ^2^(6) = 175.79, *p* < 0.001, *V* = 0.205. Adjusted residuals were used to identify the specific differences between gender identity and social profile (see Supplementary Table S2). In profile SA there were proportionately more females relative to males and other specified genders (i.e., other, prefer not to say, transgender, nonbinary, I do not know, gender fluid and gender binary). In the GSD profile, there were proportionately more males and genders not otherwise specified relative to females. The VED social profile had proportionately more females and fewer males, and lastly the VEA social profile had proportionately more males than females and genders not otherwise specified.

### Exploratory measures ANOVA

#### Duke misophonia symptom severity composite score

**Figure 4:**
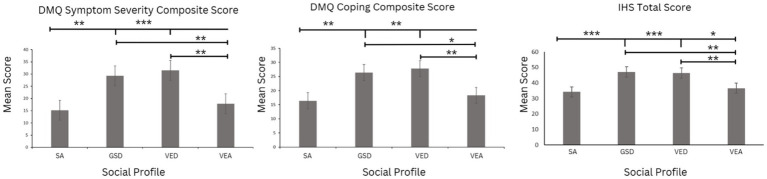
DST measured by the DMQ symptom severity composite score, DMQ coping composite score, and the IHS total score across the social profiles. Error bars indicate standard error of the mean. The magnitude of the effect is denoted with asterisks identifying comparisons that are of moderate effect size or larger, *ηp^2^ > 0.01, **ηp^2^ ≥ 0.06, ***ηp^2^ ≥ 0.14.

The Misophonia symptom severity composite scores (Figure 4) differed significantly across the social profiles, *F*(3, 2091) = 102.67 *p < 0.05*, *ηp*^2^ = 0.128. Games-Howell post-hoc analyses revealed that the DMQ symptom severity composite scores differed across all social profiles except SA and VEA, and GSD and VED profiles based on effect size (*d* > 0.2).

#### Misophonia coping composite score

The DMQ coping composite scores (Figure 4) also showed significant differences across the social profiles, *F*(3, 2091) = 57.68, *p < 0.001*, *ηp*^2^ = 0.076. Games-Howell post-hoc analyses indicated that DMQ coping composite scores were significantly different across all social profiles except SA and VEA, and GSD and VED profiles based on effect size (*d* > 0.2).

Additionally, to further assess the relationship between DST and the social profiles we analyzed the distribution of individuals who met the clinical cutoff (*n* = 378) on the DMQ across the social profiles. A chi-square test of independence showed meaningful differences, χ^2^(3) = 158.83, *p* < 0.001, *V* = 0.275. Adjusted residuals were used to identify the specific differences between clinical and non-clinical misophonia status and social profile (see Supplementary Table S3). We found a proportionately greater number of individuals with clinically severe misophonia were classified by the GSD and VED social profiles. In comparison, individuals who did not meet the cutoff for clinically significant misophonia were more often classified by the SA and VEA social profiles. These results are consistent with our findings that individuals with lower severity scores of DST, are categorized in the social profiles with the higher mean social competence scores (i.e., SA and VEA profiles; Table 3).

**Table 3:** Number of individuals meeting the clinically significant cutoff for the DMQ, across the four social profiles, compared to the number of individuals not meeting the clinically significant cut-off.

Social profile	Participants with clinically significant misophonia symptoms	Participants without clinically significant misophonia symptoms
SA	58 (0.15)	604 (0.35)
GSD	98 (0.26)	263 (0.15)
VED	163 (0.43)	329 (0.19)
VEA	59 (0.16)	521 (0.30)
Total	378	1717

Table is formatted as Number of Participants (Proportion of Participants).

#### The inventory of hyperacusis total score

IHS total scores (Figure 4) significantly differed across the social profiles, *F*(3, 2091) = 109.12, *p* < 0.001, *ηp*^2^ = 0.135. Games-Howell post-hoc analyses indicate that the main effect was driven by IHS scores that were significantly different across all social competence profiles, except GSD and VED social profiles, based on effect size (*d* > 0.20).

Additionally, to further assess the relationship between DST and the social profiles we analyzed the distribution of individuals with clinically severe levels of hyperacusis (*n* = 344) on the IHS across the social profiles. A chi-square test of independence showed meaningful differences, χ^2^(3) = 169.47, *p* < 0.001, *V* = 0.284. Adjusted residuals were used to identify the specific differences between clinical and non-clinical hyperacusis status and social profile (see Supplementary Table S4). Similarly to the DMQ results, we found a greater proportion of individuals with clinical levels of hyperacusis were classified by the GSD and VED social profiles. In comparison to the individuals with non-clinical hyperacusis, who were more frequently classified by the SA and VEA social profiles. These results are consistent with our findings that individuals with the lower severity scores of DST, are categorized in the social profiles with the higher mean social competence scores (i.e., SA and VEA; Table 4).

**Table 4:** Number of individuals meeting the clinically significant cutoff for the IHS, across the four social profiles, compared to the number of individuals not meeting the clinically significant cutoff.

Social profile	Number of participants with clinically significant hyperacusis symptoms	Number of participants without clinically significant hyperacusis symptoms
SA	40 (0.12)	622 (0.36)
GSD	111 (0.32)	250 (0.14)
VED	136 (0.40)	356 (0.20)
VEA	57 (0.17)	523 (0.30)
Total	344	1751

Table is formatted as Number of Participants (Proportion of Participants).

## Discussion

There are many factors that contribute to the development and expression of social competence. The first aim of the current study was to determine whether patterns of social processing abilities and/or difficulties tend to co-occur across individuals, allowing social profiles to be established. The second aim was to examine whether DST severity varied as a function of these social profiles to determine whether DST may be related to social competence development and expression.

### Social profiles

Using k-means cluster analyses, we identified four unique social profiles that parsed variability in social strengths and challenges across seven social competence domains. The first social profile, identified is a socially adaptive profile (SA), describes individuals who demonstrate strengths across all seven social competence domains. The second social profile, a generalized social differences profile (GSD), consists of individuals who experience challenges across all seven social domains measured. The SA and GSD profiles represent the opposite extremes of social processing abilities and highlight the fact that there are large individual differences in social abilities, even among a sample of undergraduate university students. Two additional intermediate profiles were identified, further highlighting the heterogeneity in social processing abilities. The third social profile, the verbal and emotion regulation differences profile (VED), consists of people who appear to have distinct challenges with verbal conversation skills and emotion regulation. Finally, the fourth social profile, the verbal and emotion regulation adaptive profile (VEA), contains individuals with social motivation, empathy, and non-verbal communication difficulties, but markedly strong verbal conversation skills and emotion regulation abilities.

A closer look at the intermediate profiles, VEA and VED, highlights the benefit of this social profile approach to understanding variability in social abilities. Individuals characterized by the VEA and VED profiles had similar total scores on the MSCS, yet these individuals have very different abilities to demonstrate empathetic concern, communicate verbally and regulate their emotions. Without taking this more nuanced approach, these differences may be overlooked, missing the opportunity to target more specific social strengths and challenges. In clinical settings, these four social profiles may allow for a more comprehensive depiction of individuals’ social abilities and may support more tailored approaches to social skills support. In the context of research, these social profiles provide a novel way for understanding and parsing the heterogeneity in social behaviors. By understanding the nuances of social differences, researchers will be able to measure and control aspects of the variability in social abilities, thus aiding their ability to investigate relations with other factors, as we were able to do here.

To further characterize the individuals described by these four social profiles, we examined potential differences in demographic characteristics across the profiles. Age was not found to vary across the profiles, although this was not particularly surprising given the sample was undergraduate students. Upon examination of the distribution of gender across the social profiles, the SA and VED profiles tended to have proportionately more females, while males were more common in the VEA profile, and both males and gender not otherwise specified participants were more frequently classified by the GSD profile, relative to the expected gender distribution of the sample. This suggests that gender may also play a role in shaping an individual’s social abilities, contributing to an individual’s social profile. Future research should further investigate the relationship between social abilities and gender identity as there may be significant societal implications for better understanding the interplay between social profile and gender identity.

### Relating social profiles to DST characteristics

Relations between social and sensory processing have been previously established (Hilton et al., 2007; Kose et al., 2023; Scheerer et al., 2021), and DST has been shown to promote social avoidance (Dibb and Golding, 2022; Scheerer et al., 2021; Smees et al., 2024; Blaesing and Kroener-Herwig, 2012). Moreover, Dibb and Golding (2022), found that longitudinally, individuals with misophonia reported persistent avoidance behaviors related to their misophonic triggers and severity. Thus, given the previously established relationships between DST and social processes the second aim of the current study was to investigate a potential relationship between DST symptoms and social competence. Both misophonia symptom severity and coping abilities, as well as hyperacusis severity were found to vary across the four social profiles. Notably, people characterized by the SA profile, those with the most adaptive social abilities, also had the fewest DST symptoms. The highest levels of DST symptoms were found in those classified by the GSD and VED social profiles. As those classified by the GSD and VED social profiles had the lowest total social competence scores, this suggests an association between higher DST severity and lower social competence. Furthermore, this is supported by correlational analyses which found that most MSCS subscales were significantly negatively related to DST severity measures. These findings are in line with previous work that suggests sensory processing differences have a significant influence on the variation and expression of social abilities (Scheerer et al., 2021; Yager and Iarocci, 2013). However, the goal of the current work was to go beyond comparing DST symptoms to global differences in social abilities. As such, considering the commonalities among the GSD and VED social profiles, verbal conversation skills and emotion regulation are the notable difficulties across both profiles. Thus, this work builds on previous associations between sensory and social processing by describing a more specific relation between DST and differences in verbal conversation skills and emotional regulation.

These social profiles offer a framework for understanding how varying levels of DST distinctly impact the development and expression of social competence. Individuals with more severe DST were more frequently classified within the VED and GSD profiles—groups characterized by shared social difficulties such as challenges in social inferencing, verbal communication, and emotion regulation. In contrast, those with less severe DST were more commonly represented in the SA and VEA profiles. These patterns suggest that DST severity shapes the social landscape differently across profiles, pointing to distinct areas of strength and vulnerability. These findings underscore the potential benefit of modifying sensory environments—particularly those involving auditory stimuli—to better support individuals with DST. Rather than targeting the individual for change, tailoring environments to reduce exposure to triggering sounds (e.g., through acoustic design, noise reduction strategies, or sensory-friendly settings) may help mitigate the negative impact of DST on social engagement and functioning. For instance, the observation that individuals with clinical levels of hyperacusis and misophonia often fell into the VED profile highlights the importance of addressing sensory stressors as a pathway to improving social outcomes. These insights can guide the development of context-sensitive interventions in clinical, educational, and community settings, ultimately promoting more equitable opportunities for social interaction among those with DST.

Misophonia and hyperacusis have been described as independent DST conditions (Williams et al., 2021). However, in our current study, DMQ and IHS scores showed similar patterns of severity across the established social profiles. It is possible that despite unique underlying physiological mechanisms, misophonia and hyperacusis may have similar effects on the development and expression of social behaviors. For example, both misophonia and hyperacusis are thought to promote avoidance of environments with potentially triggering and/or distressing sounds (Scheerer et al., 2021; Smees et al., 2024; Blaesing and Kroener-Herwig, 2012). Given many social environments (e.g., parks, restaurants, movie theaters, etc.) are sensory rich, both misophonia and hyperacusis are likely to impact social experiences in a similar manner. Further, many people with misophonia and hyperacusis use noise canceling devices (e.g., headphones, ear plugs, etc.) to block out potentially triggering sounds (Scheerer et al., 2024a,b,c; Wunrow, 2024) While this strategy may help individuals avoid sound triggers, it also limits exposure to sounds in general, including an important social sound, speech. This reduced social contact and speech exposure, respectively, may underlie differences in the development and expression of verbal conversation skills. Similarly, both misophonia and hyperacusis have been reported to evoke strong emotional responses (Scheerer et al., 2021; Aazh et al., 2019; Henry et al., 2022). As such, it is possible that the heightened sensitivity to sounds creates additional challenges for emotional regulation with the intense emotional responses induced. Thus, the relations between DST and social competence may be driven by more generalized neurological sensory processing differences rather than condition-specific difficulties. This finding is important, as it suggests that even if misophonia and hyperacusis have different etiologies, they may share similar responses and coping mechanisms and as a result they may have similar relations to other affected functions like social development and expression.

The relationship between DST and social competence suggests that those affected by DST may have poorer social outcomes. Given social functioning plays such an integral part of our lives as humans, this could have further downstream effects on things like academic outcomes, job success, mental health, and many other behaviors that may rely on social competence (Smith et al., 2023; Seeber and Wittmann, 2017; Romppanen et al., 2021; Tabassum et al., 2020). Therefore, further research to explore this relationship between DST and social functioning is essential to help mitigate the adverse effects of this relationship, support individuals with DST, and provide accommodations.

With regards to post-secondary environments, sensory differences can create significant barriers to socialization, academic success, and housing satisfaction while students are away at school (Dwyer et al., 2023). However, finding the right accommodations for those with DST may be difficult, because if designed incorrectly, accommodations that support sensory differences may also potentially negatively influence social outcomes. A recent publication written by a group of neurodivergent undergraduate and graduate students, along with alumni and parent allies, suggests recommendations for accommodations and promoting inclusion for neurodiverse students, staff and faculty in post-secondary institutions, particularly with regards to their sensory differences (Dwyer et al., 2023). While the respondents in the current study were not all neurodivergent, given many of the recommendations by Dwyer et al. (2023) focused on supporting sensory differences, they are quite relevant to the current discussion. As such, it was recommended that students should have access to spaces free from overwhelming sensory information. For example, offering students the option of a single room with no roommates in a building with strict noise regulations would be particularly beneficial to students with sensory difficulties, including DST. Although not having a roommate may reduce social contact, having a sensory-safe environment to decompress may reduce overall levels of distress, allowing those with DST to engage in other social engagements/environments. Another recommendation specifically relevant to DST is within dining halls. Dining areas expose students to various sounds and foods that may be very triggering to individuals with sensory differences, especially those with misophonia (Swedo et al., 2022). As such, alternatives to sensory-aversive foods should be present for individuals, providing many sensory-safe meal options (Dwyer et al., 2023). Additionally, outdoor eating spaces allow students to take food outside to avoid exposure to trigger sounds. This may alleviate distress created by eating/food-related triggers, but more importantly, ensure that students are not experiencing malnutrition due to reduced accessibility in dining facilities. Specific to academic outcomes, triggering noises/noise levels have the potential to influence information retention during lectures and performance during quizzes and exams for students with sensory differences. Even everyday noise sources like air conditioners or lights can have adverse effects on academic outcomes for those with DST. Therefore, for the success of individuals with sensory differences, having carefully designed and engineered spaces where these distractions are reduced is imperative (Dwyer et al., 2023), particularly so that students can attend classes and interact with peers without experiencing high levels of distress.

Given the established association between social competence and DST, another potential approach to supporting those with DST could be interventions aimed at improving social competence in individuals with DST. However, our framing of DST is grounded in a neurodiversity-affirming perspective, which emphasizes variation rather than deficit (Gurba et al., 2024; Scheerer et al., 2024a,b,c). In line with this approach, we are cautious about endorsing interventions that place the onus on individuals to adapt, particularly when social difficulties may stem from environmental barriers or normative expectations rather than inherent impairments (Graf-Kurtulus and Gelo, 2025). Therefore, we emphasize the importance of modifying social and educational environments to be more inclusive of diverse sensory preferences as well as social styles. We believe this is a more ethically aligned and socially responsive approach to supporting individuals with DST.

The relations between higher levels of DST and poorer social competence revealed in the current study emphasize the need to focus on finding and prioritizing accommodations to support individuals who suffer from DST so that they can thrive and achieve their personal, academic, and occupational goals. Recently, efforts to develop consensus definitions of misophonia (Swedo et al., 2022) and hyperacusis (Adams et al., 2020), as well as to develop validated diagnostic tools (e.g., Rosenthal et al., 2021; Williams et al., 2022; Carson et al., 2023) have rapidly accelerated research on DST. However, future research needs to continue to leverage this information and these tools to better understand how DST develops, changes throughout the lifespan, and influences other aspects of functioning (Carson et al., 2023; Williams et al., 2022). In time, we may develop solutions to help mitigate the adverse effects of DST, including finding ways to reduce avoidance behaviors, withdrawal from social participation, and ideally improve overall well-being for those with DST.

### Limitations

It is essential to recognize the limitations of any research study. The results of this study were based on subjective self-report measures of social competence, misophonia and hyperacusis. This may have limited the objectivity and reliability of the data, as well as introduced bias to the responses as individuals may lack self-awareness or may misinterpret items on the questionnaires. Additionally, our sample consisted of university students in Southern Ontario, which limits the generalizability as the sample consists of a largely homogenous group of young adults, most of whom do not have clinically severe DST. We also did not collect information about the socio-economic status of these students; thus, we were unable to assess the possible influence of this variable. There was also an uneven gender distribution in our sample, as there were more female participants than any other gender. Thus, although we report gender differences across our social profiles, further work with more gender-balanced samples is required to validate these findings.

Additionally, when conducting a *k* means cluster analysis, it is typical that the *k* cluster solution chosen has the lowest BIC value. However, in the current study we did not use the *k* cluster solution with the lowest BIC value. The silhouette scores were also evaluated, and the four-cluster model had the second-best silhouette score, after the two-cluster model. The four-cluster model was selected as it was more interpretable regarding the clusters themselves with the data. The four-cluster model produced more visually distinguishable social profiles than the five-cluster solution. Additionally, an elbow point was found on the graph of the BIC values and the WCSS, which depicts a slowed rate of improvement starting with the four-cluster solution, meaning that there was not a large difference between the *k* 4 and *k* 5 BIC or WCSS values. This meant that the five-cluster solution provided only a slightly better statistical model yet a less visually interpretable one.

## Conclusion

Overall, our results demonstrate a significant relationship between DST and social competence, with individuals with more severe DST demonstrating lower total social competence scores. However, the social profiles from our cluster analyses provided more insight into the specific challenges individuals with more severe DST may face. A greater number of individuals with higher severity scores of DST were in the GSD and VED social profiles. Challenges with verbal conversation skills and emotion regulation characterize these social profiles, which may allude to specific challenges with social competence development and expression due to DST. These results also emphasize the need to support individuals with DST, as these results suggest that DST may have adverse downstream effects on social abilities.

## Data Availability

The raw data supporting the conclusions of this article will be made available by the authors, without undue reservation.
