# Supplementary material for: Social success in a noisy world: exploring the relationship between decreased sound tolerance and social profiles
Source: Front Psychol. 2025 May 22;16:1560100. doi: 10.3389/fpsyg.2025.1560100 (PMC12139024; doi:10.3389/fpsyg.2025.1560100)
Supplement: Supplementary file 1 [file Data_Sheet_1.pdf]

Supplemental Materials

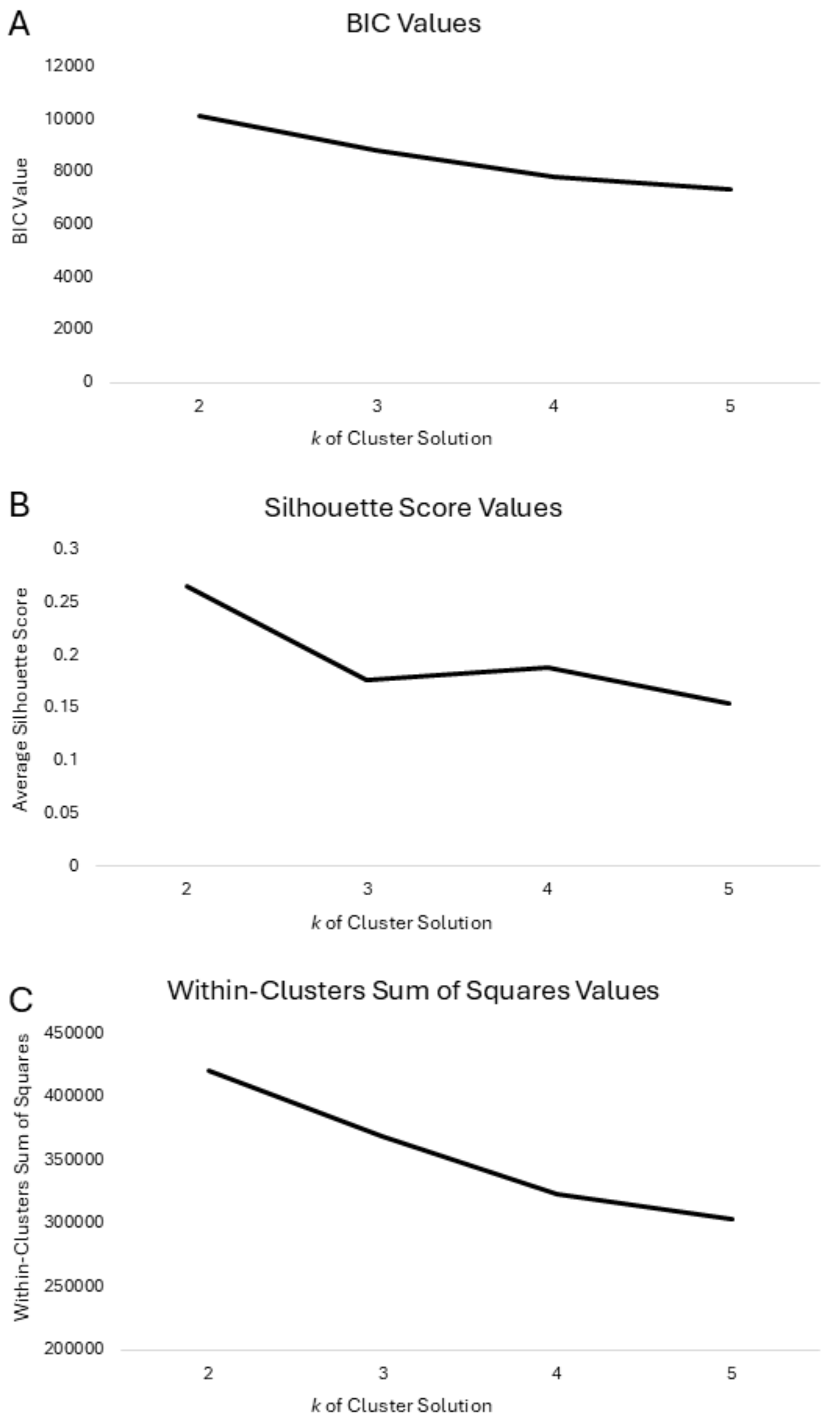

Figure S1. (A) Bayesian Information Criterion (BIC) values, (B) average silhouette scores, and (C) Within-Cluster Sum of Squares (WCSS) of the *k* 2 to *k* 5 cluster solutions.

Table S1. First order correlations between MSCS subscales, DMQ Symptom Severity, DMQ Coping, and IHS Total Score. Given DMQ Symptom Severity and Coping scores as well as IHS total score all violated assumptions of normality, a non-parametric Spearman's rho was calculated.

		DMQ Coping	DMQ Symptom Severity	IHS Total Score
SI	rho	-0.201	-0.228	-0.257
	p	< .001	< .001	< .001
SM	rho	-0.193	-0.245	-0.265
	p	< .001	< .001	< .001
DEM	rho	0.034	0.011	-0.006
	p	0.124	0.606	0.778
SK	rho	-0.114	-0.125	-0.154
	p	< .001	< .001	< .001
VCS	rho	-0.288	-0.325	-0.329
	p	< .001	< .001	< .001
NCS	rho	-0.144	-0.146	-0.186
	p	< .001	< .001	< .001
ER	rho	-0.349	-0.471	-0.445
	p	< .001	< .001	< .001

Table S2. Chi-square test of independence for gender and social profile. Note * indicating Z scores ±1.96 that demonstrate statistically significant differences.

		Male	Female	Not Otherwise Specified
SA				
	Count	92*	565*	5*
	Percent	13.9%	85.3%	0.8%
	Expected Count	138	510	14
	Adjusted Residual	-5.3	6.1	-3.0
GSD				
	Count	96*	245*	20*
	Percent	26.6%	67.9%	5.5%
	Expected Count	75	278	8
	Adjusted Residual	3.0	-4.6	4.9
VED				
	Count	43*	435*	14
	Percent	8.7%	88.4%	2.8%
	Expected Count	102	379	11
	Adjusted Residual	-7.5	6.9	1.2

VEA				
	Count	205*	369*	6*
	Percent	35.3%	63.6%	1.0%
	Expected Count	121	447	12
	Adjusted Residual	10.1	-9.0	-2.2

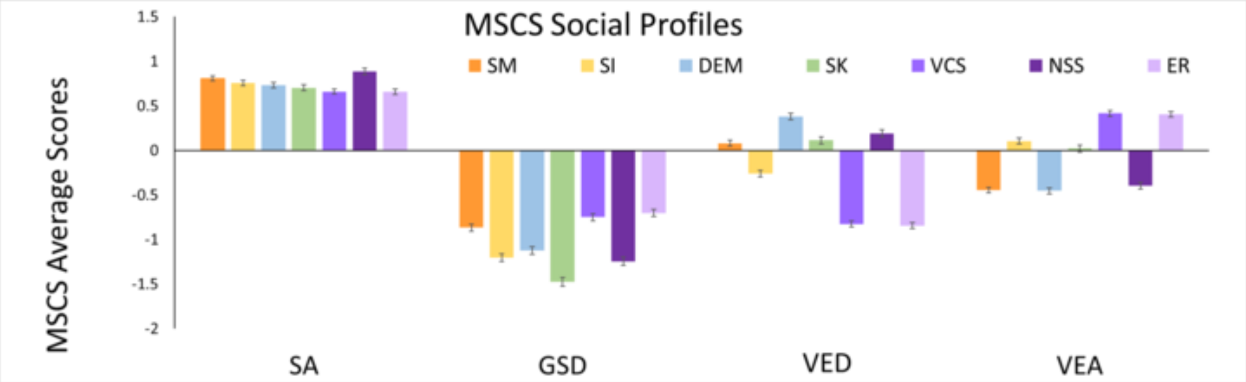

Figure S2. Social competence profiles based on z-scores of the MSCS across seven subscales, and a k 4 cluster solution. Negative z-scores indicate increased social competence difficulties. Error bars indicate standard error of the z-scores.

Table S3. Chi-square test of independence for clinical/sub-clinical misophonia status and social profile. Note profiles with * demonstrate statistically significant differences relative to profiles with ^ (Z scores ±1.96).

		Sub-Clinical	Clinical
SA			
	Count	604*	58*
	Percent	28.8%	2.8%
	Expected Count	543	119
	Adjusted Residual	7.5	-7.5
GSD			
	Count	263^	98^
	Percent	12.6%	4.7%
	Expected Count	296	65
	Adjusted Residual	-4.9	4.9
VED			
	Count	329^	163^
	Percent	15.7%	7.8%
	Expected Count	403	89
	Adjusted Residual	-10.0	10.0

VEA			
	Count	521*	59*
	Percent	24.9%	2.8%
	Expected Count	475	105
	Adjusted Residual	5.8	-5.8

Table S4. Chi-square test of independence for clinical/sub-clinical hyperacusis status and social profile. Note profiles with * demonstrate statistically significant differences relative to profiles with ^ (Z scores ±1.96).

		Male	Female
SA			
	Count	622*	40*
	Percent	29.7%	1.9%
	Expected Count	533	109
	Adjusted Residual	8.7	-8.7
GSD			
	Count	250^	111^
	Percent	11.9%	5.3%
	Expected Count	302	59
	Adjusted Residual	-8.1	8.1
VED			
	Count	356^	136^
	Percent	17.0%	6.5%
	Expected Count	102	379
	Adjusted Residual	-7.7	7.7
VEA			
	Count	523*	57*
	Percent	25.0%	2.7%
	Expected Count	485	95
	Adjusted Residual	5.0	-5.0
